# Dibromidodimethyl­dipyridine­platinum(IV)

**DOI:** 10.1107/S160053680803208X

**Published:** 2008-10-11

**Authors:** Mairéad E. Kelly, Christoph Wagner, Harry Schmidt

**Affiliations:** a Institut für Chemie, Kurt-Mothes-Straße 2, Martin-Luther-Universität Halle-Wittenberg, 06120 Halle, Germany

## Abstract

In the title complex, [PtBr_2_(CH_3_)_2_(C_5_H_5_N)_2_], the Pt^IV^ metal centre lies on a twofold rotation axis and adopts a slightly distorted octa­hedral coordination geometry. The structure displays weak intra­molecular C—H⋯Br hydrogen-bonding inter­actions.

## Related literature

For the crystal structures of related compounds, see: Brammer *et al.* (2001 ▶); Burton *et al.* (1983 ▶); Canty *et al.* (1990 ▶); Clark *et al.* (1983 ▶); Contreras *et al.* (2001 ▶); Hall & Swile (1971 ▶); Hindmarch *et al.* (1997 ▶); Hughes *et al.* (2001 ▶); Kaluderović *et al.* (2007 ▶); Kelly, Gómez-Ruiz, Kluge *et al.* (2008 ▶); Kelly, Gómez-Ruiz, Schmidt *et al.* (2008 ▶); Kelly, Dietrich *et al.* (2008 ▶); Klingler *et al.* (1982 ▶). For bond-length data, see: Allen (2002 ▶).
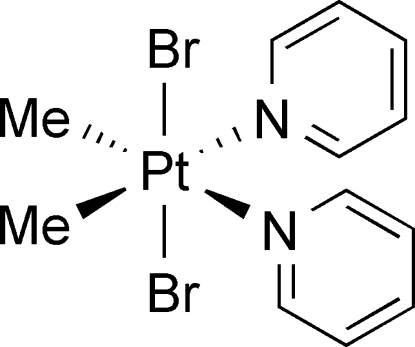


## Experimental

### 

#### Crystal data

[PtBr_2_(CH_3_)_2_(C_5_H_5_N)_2_]
*M* *_r_* = 543.18
Orthorhombic, 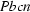

*a* = 13.297 (2) Å
*b* = 8.2906 (15) Å
*c* = 13.516 (3) Å
*V* = 1490.1 (5) Å^3^
*Z* = 4
Mo *K*α radiation
μ = 14.76 mm^−1^
*T* = 220 (2) K
0.40 × 0.34 × 0.30 mm

#### Data collection

Stoe IPDS diffractometer
Absorption correction: numerical (*IPDS*; Stoe & Cie, 1999 ▶) *T*_min_ = 0.024, *T*_max_ = 0.069
10568 measured reflections
1453 independent reflections
1166 reflections with *I* > 2σ(*I*)
*R*_int_ = 0.144

#### Refinement

*R*[*F* ^2^ > 2σ(*F* ^2^)] = 0.037
*wR*(*F* ^2^) = 0.091
*S* = 1.01
1453 reflections
80 parameters
H-atom parameters constrained
Δρ_max_ = 1.71 e Å^−3^
Δρ_min_ = −1.68 e Å^−3^

### 

Data collection: *IPDS Software* (Stoe & Cie, 1999 ▶); cell refinement: *IPDS Software*; data reduction: *IPDS Software*; program(s) used to solve structure: *SHELXS97* (Sheldrick, 2008 ▶); program(s) used to refine structure: *SHELXL97* (Sheldrick, 2008 ▶); molecular graphics: *ORTEP-3* (Farrugia, 1997 ▶); software used to prepare material for publication: *SHELXL97*.

## Supplementary Material

Crystal structure: contains datablocks I, global. DOI: 10.1107/S160053680803208X/rz2250sup1.cif

Structure factors: contains datablocks I. DOI: 10.1107/S160053680803208X/rz2250Isup2.hkl

Additional supplementary materials:  crystallographic information; 3D view; checkCIF report

## Figures and Tables

**Table 1:** Hydrogen-bond geometry (Å, °)

*D*—H⋯*A*	*D*—H	H⋯*A*	*D*⋯*A*	*D*—H⋯*A*
C6—H8⋯Br^i^	0.93	2.92	3.412 (6)	115

Symmetry code: (i) 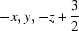.

**Fig. 1.**
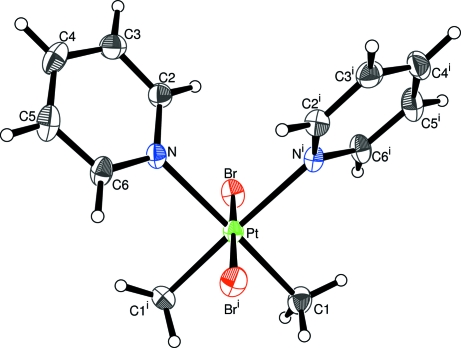
A view of the title complex with displacement ellipsoids drawn at the 30% probability level. H atoms are shown as small spheres of arbitrary radii. [Symmetry code: (i) -x, y, -z + 3/2z].

## supplementary crystallographic information

### Comment

The structure of the title compound is one of a relatively small number of structures with the PtBr_2_Me_2_ moiety (Contreras *et al.*, 2001; Kaluderović *et al.*, 2007; Kelly, Gómez-Ruiz, Kluge *et al.*, 2008; Kelly, Gómez-Ruiz, Schmidt *et al.*, 2008; Kelly, Dietrich *et al.*, 2008). The compound crystallizes in the orthorhombic space group *Pbcn* and half the molecule is generated by a twofold crystallographic axis bisecting the C—Pt—N axis as illustrated in Fig. 1. The ligating atoms have an approximate octahedral arrangement around the platinum atom. The Pt—N bond length (2.226 (5) Å) is slightly longer than expected for a platinum(IV)—N bond *trans*-configured to a ligating carbon atom (median: 2.156 Å; lower/upper quartile: 2.135/2.194 Å for 402 entries in the Cambridge Structural Database; CSD, Version 5.28, August 2007; Allen, 2002). The Pt—Br bond length (2.461 (1) Å) and the Pt—C bond length (2.053 (7) Å) are typical for bonds of these types (Clark *et al.*, 1983; Klingler *et al.*, 1982; Burton *et al.*, 1983; Hughes *et al.*, 2001; Canty *et al.*, 1990; Hindmarch *et al.*, 1997; Kelly, Gómez-Ruiz, Kluge *et al.*, 2008; Kelly, Gómez-Ruiz, Schmidt *et al.*, 2008; Kelly, Dietrich *et al.*, 2008). A short intramolecular distance between the C6 carbon atom of the pyridine ligand and a bromo ligand of the same molecule is found, indicating the presence of weak C—H···Br interactions (Brammer *et al.*, 2001).

### Experimental

The title compound was prepared by dissolving [(PtBr_2_Me_2_)*_n_*] in an excess of pyridine (Hall & Swile, 1971). Single crystals suitable for X-ray analysis were obtained by slow evaporation of a chloroform solution.

### Refinement

H atoms were positioned geometrically and treated as riding, with C—H bonding lengths constrained to 0.93–0.96 Å and with *U*_iso_(H) = 1.2*U*_eq_(C). The poor quality of the crystal may account for the rather high R_int_ value.

### Crystal data

**Table:**

[PtBr_2_(CH_3_)_2_(C_5_H_5_N)_2_]	*F*(000) = 1000
*M**_r_* = 543.18	*D*_x_ = 2.421 Mg m^−^^3^
Orthorhombic, *P**b**c**n*	Mo *K*α radiation, λ = 0.71073 Å
Hall symbol: -P 2n 2ab	Cell parameters from 8000 reflections
*a* = 13.297 (2) Å	θ = 2.2–25.9°
*b* = 8.2906 (15) Å	µ = 14.76 mm^−^^1^
*c* = 13.516 (3) Å	*T* = 220 K
*V* = 1490.1 (5) Å^3^	Block, orange
*Z* = 4	0.40 × 0.34 × 0.30 mm

### Data collection

**Table:**

Stoe IPDS diffractometer	1453 independent reflections
Radiation source: fine-focus sealed tube	1166 reflections with *I* > 2σ(*I*)
graphite	*R*_int_ = 0.144
area detector scans	θ_max_ = 26.0°, θ_min_ = 2.9°
Absorption correction: numerical (IPDS; Stoe & Cie, 1999)	*h* = −16→16
*T*_min_ = 0.024, *T*_max_ = 0.069	*k* = −10→10
10568 measured reflections	*l* = −16→16

### Refinement

**Table:**

Refinement on *F*^2^	Secondary atom site location: difference Fourier map
Least-squares matrix: full	Hydrogen site location: inferred from neighbouring sites
*R*[*F*^2^ > 2σ(*F*^2^)] = 0.037	H-atom parameters constrained
*wR*(*F*^2^) = 0.091	*w* = 1/[σ^2^(*F*_o_^2^) + (0.0472*P*)^2^] where *P* = (*F*_o_^2^ + 2*F*_c_^2^)/3
*S* = 1.01	(Δ/σ)_max_ = 0.002
1453 reflections	Δρ_max_ = 1.71 e Å^−^^3^
80 parameters	Δρ_min_ = −1.68 e Å^−^^3^
0 restraints	Extinction correction: SHELXL97 (Sheldrick, 2008), Fc^*^=kFc[1+0.001xFc^2^λ^3^/sin(2θ)]^-1/4^
Primary atom site location: structure-invariant direct methods	Extinction coefficient: 0.0012 (2)

### Special details

**Table:**

Geometry. All e.s.d.'s (except the e.s.d. in the dihedral angle between two l.s. planes) are estimated using the full covariance matrix. The cell e.s.d.'s are taken into account individually in the estimation of e.s.d.'s in distances, angles and torsion angles; correlations between e.s.d.'s in cell parameters are only used when they are defined by crystal symmetry. An approximate (isotropic) treatment of cell e.s.d.'s is used for estimating e.s.d.'s involving l.s. planes.
Refinement. Refinement of *F*^2^ against ALL reflections. The weighted *R*-factor *wR* and goodness of fit *S* are based on *F*^2^, conventional *R*-factors *R* are based on *F*, with *F* set to zero for negative *F*^2^. The threshold expression of *F*^2^ > σ(*F*^2^) is used only for calculating *R*-factors(gt) *etc*. and is not relevant to the choice of reflections for refinement. *R*-factors based on *F*^2^ are statistically about twice as large as those based on *F*, and *R*- factors based on ALL data will be even larger.

### Fractional atomic coordinates and isotropic or equivalent isotropic displacement parameters (Å^2^)

**Table:**

	*x*	*y*	*z*	*U*_iso_*/*U*_eq_	
C1	0.0667 (6)	−0.0872 (9)	0.6693 (6)	0.0487 (17)	
H3	0.1207	−0.1336	0.7069	0.058*	
H1	0.0928	−0.0434	0.6087	0.058*	
H2	0.0179	−0.1690	0.6544	0.058*	
C2	−0.1238 (5)	0.4045 (8)	0.7975 (5)	0.0412 (15)	
H4	−0.1268	0.4089	0.7288	0.049*	
C3	−0.1703 (5)	0.5237 (9)	0.8514 (6)	0.0479 (17)	
H5	−0.2038	0.6070	0.8191	0.057*	
C4	−0.1673 (5)	0.5196 (10)	0.9542 (6)	0.0526 (19)	
H6	−0.1976	0.6001	0.9918	0.063*	
C5	−0.1182 (5)	0.3930 (10)	0.9987 (6)	0.057 (2)	
H7	−0.1161	0.3847	1.0673	0.068*	
C6	−0.0716 (4)	0.2771 (9)	0.9396 (4)	0.0423 (15)	
H8	−0.0373	0.1928	0.9699	0.051*	
N	−0.0747 (3)	0.2833 (7)	0.8400 (3)	0.0355 (11)	
Br	−0.14288 (5)	0.08616 (9)	0.63441 (5)	0.0465 (2)	
Pt	0.0000	0.09312 (4)	0.7500	0.03167 (18)	

### Atomic displacement parameters (Å^2^)

**Table:**

	*U*^11^	*U*^22^	*U*^33^	*U*^12^	*U*^13^	*U*^23^
C1	0.055 (4)	0.047 (4)	0.044 (4)	−0.004 (3)	0.007 (3)	−0.012 (3)
C2	0.039 (3)	0.045 (4)	0.039 (4)	0.002 (3)	−0.001 (3)	0.003 (3)
C3	0.036 (3)	0.044 (4)	0.064 (5)	0.000 (3)	0.004 (3)	0.003 (4)
C4	0.035 (3)	0.062 (5)	0.061 (5)	−0.001 (3)	0.010 (3)	−0.023 (4)
C5	0.045 (4)	0.087 (7)	0.038 (4)	0.002 (3)	0.006 (3)	−0.010 (4)
C6	0.034 (3)	0.063 (4)	0.030 (3)	0.007 (3)	−0.002 (2)	−0.004 (3)
N	0.029 (2)	0.047 (3)	0.030 (3)	0.000 (2)	0.0029 (19)	0.003 (2)
Br	0.0385 (3)	0.0661 (5)	0.0347 (4)	−0.0103 (3)	−0.0087 (3)	0.0010 (3)
Pt	0.0312 (2)	0.0397 (2)	0.0242 (3)	0.000	−0.00189 (11)	0.000

### Geometric parameters (Å, °)

**Table:**

C1—Pt	2.053 (7)	C4—H6	0.9300
C1—H3	0.9600	C5—C6	1.394 (10)
C1—H1	0.9600	C5—H7	0.9300
C1—H2	0.9600	C6—N	1.347 (7)
C2—N	1.329 (8)	C6—H8	0.9300
C2—C3	1.375 (10)	N—Pt	2.226 (5)
C2—H4	0.9300	Br—Pt	2.4605 (7)
C3—C4	1.391 (10)	Pt—C1^i^	2.053 (7)
C3—H5	0.9300	Pt—N^i^	2.226 (5)
C4—C5	1.375 (11)	Pt—Br^i^	2.4605 (7)
			
Pt—C1—H3	109.5	C5—C6—H8	118.9
Pt—C1—H1	109.5	C2—N—C6	118.4 (6)
H3—C1—H1	109.5	C2—N—Pt	121.2 (4)
Pt—C1—H2	109.5	C6—N—Pt	120.4 (5)
H3—C1—H2	109.5	C1—Pt—C1^i^	86.5 (4)
H1—C1—H2	109.5	C1—Pt—N	178.4 (2)
N—C2—C3	122.4 (7)	C1^i^—Pt—N	91.9 (3)
N—C2—H4	118.8	C1—Pt—N^i^	91.9 (3)
C3—C2—H4	118.8	C1^i^—Pt—N^i^	178.4 (2)
C2—C3—C4	119.9 (7)	N—Pt—N^i^	89.8 (3)
C2—C3—H5	120.0	C1—Pt—Br^i^	89.2 (2)
C4—C3—H5	120.0	C1^i^—Pt—Br^i^	88.8 (2)
C5—C4—C3	117.9 (7)	N—Pt—Br^i^	90.80 (12)
C5—C4—H6	121.0	N^i^—Pt—Br^i^	91.11 (12)
C3—C4—H6	121.0	C1—Pt—Br	88.8 (2)
C4—C5—C6	119.1 (7)	C1^i^—Pt—Br	89.2 (2)
C4—C5—H7	120.4	N—Pt—Br	91.11 (12)
C6—C5—H7	120.4	N^i^—Pt—Br	90.80 (12)
N—C6—C5	122.2 (7)	Br^i^—Pt—Br	177.31 (4)
N—C6—H8	118.9		
			
N—C2—C3—C4	−0.3 (10)	C5—C6—N—Pt	−179.4 (5)
C2—C3—C4—C5	−0.9 (11)	C2—N—Pt—N^i^	49.9 (4)
C3—C4—C5—C6	1.6 (11)	C6—N—Pt—N^i^	−130.6 (6)
C4—C5—C6—N	−1.3 (11)	C2—N—Pt—Br^i^	141.0 (4)
C3—C2—N—C6	0.7 (9)	C6—N—Pt—Br^i^	−39.5 (5)
C3—C2—N—Pt	−179.8 (5)	C2—N—Pt—Br	−40.9 (4)
C5—C6—N—C2	0.1 (10)	C6—N—Pt—Br	138.6 (5)

Symmetry codes: (i) −*x*, *y*, −*z*+3/2.

### Hydrogen-bond geometry (Å, °)

**Table:**

*D*—H···*A*	*D*—H	H···*A*	*D*···*A*	*D*—H···*A*
C6—H8···Br^i^	0.93	2.92	3.412 (6)	115

Symmetry codes: (i) −*x*, *y*, −*z*+3/2.
